# Manual wheelchair training approaches and intended training outcomes for adults who are new to wheelchair use: A scoping review

**DOI:** 10.1111/1440-1630.12992

**Published:** 2024-10-01

**Authors:** Kimberly Charlton, Carolyn Murray, Natasha Layton, Emilee Ong, Lucy Farrar, Trish Serocki, Stacie Attrill

**Affiliations:** ^1^ School of Allied Health Science and Practice University of Adelaide Adelaide Australia; ^2^ School of Allied Health and Human Performance University of South Australia Adelaide Australia; ^3^ Rehabilitation, Ageing and Independent Living Research Centre Monash University Melbourne Australia

**Keywords:** aged care, chronic conditions, education, International Classification of Functioning (ICF), manual wheelchair training

## Abstract

**Introduction:**

Wheelchair training is pivotal for safety, independence, and occupational engagement in the community, yet adults coming into wheelchair use often receive insufficient or untailored training. This research aimed to understand the range and type of manual wheelchair training approaches that exist for adults commencing wheelchair use.

**Method:**

A systematic scoping review involved searching eight electronic databases and grey literature up to September 2023. Papers relating to manual wheelchair training for adults and their caregivers were included for data extraction. Eighty‐seven articles were included in this review. The International Classification of Functioning (ICF) was used to organise and analyse data related to intended training outcomes.

**Consumer and Community Involvement:**

Consumer consultation was not included in this review; however, the outcomes suggest that involving consumers in future wheelchair training research is critical to assure community participation outcomes.

**Results:**

Data were extracted from 87 papers. Manual wheelchair training was delivered across diverse contexts encompassing varied support structures, trainer backgrounds, and technology and was commonly directed towards wheelchair users with spinal cord injury. Intended training outcomes most frequently mapped to the activity and participation component of the ICF (*n* = 39), followed by personal factors (*n* = 27), body structures and functions (*n* = 18), and environmental factors (*n* = 3), with limited focus on longer term occupational engagement outcomes.

**Conclusion:**

Most existing manual wheelchair training focussed on the acquisition of individual wheelchair skill and may not facilitate generalised and long‐term occupational participation outcomes. Further exploration into the contexts that support occupational engagement, particularly for older adults with progressive conditions, is required to support service provision.

**PLAIN LANGUAGE SUMMARY:**

We looked at what manual wheelchair training approaches exist for adults who need to use a manual wheelchair and what training helps people to do/achieve. We did a scoping review that looked at literature about manual wheelchair training programs for adult wheelchair users. We found 87 research papers and training programs that we included in our review. We recorded and analysed information from all the papers about the wheelchair training programs and outcomes for people who do these programs. We found that manual wheelchair training can be done in structured or ad hoc ways, can have different amounts of training, can be provided face‐to‐face or online, and can be given by different allied health professionals and other wheelchair users. Most training programs had short‐term outcomes like learning manual wheelchair skills, being able to use the wheelchair properly, and feeling confident about using a wheelchair. Some had longer term outcomes about being able to use the manual wheelchair in everyday activities. Most people who did the training programs that we looked at in this review were manual wheelchair users with spinal cord injury. Because not many wheelchair programs have been tried with people who do not have a spinal cord injury, it is hard for occupational therapists to make recommendations about training for other people who use a manual wheelchair. Manual wheelchair training that is done in the community and made to meet the needs of individuals may help people with using their wheelchair for their everyday activities and participate in their community.

**Key Points for Occupational Therapy**

- Few manual wheelchair training programs are tailored to client context, their personal and environmental factors and individualised activity and participation goals.
- An occupational focus is required when delivering manual wheelchair training.
- Knowing more about longer term occupational participation outcomes will improve decisions about wheelchair training.

## 1 INTRODUCTION

Wheelchair provision is considered a basic human right and supports independence in activities of daily living and community access (WHO, 2023). Independence arising from wheelchair provision reduces reliance on carers/families, supports manual wheelchair (MWC) users to stay living at home longer, and provides a means for social interaction and meaningful occupational engagement, which is key in supporting psychological and social wellbeing (WHO, 2023).

An important component of MWC provision is the delivery of appropriate training (WHO, 2023). Although MWC training can fall under the scope of wheelchair provision, many MWC training programs are situated independently from wheelchair provision (Tu et al., 2017). This practice is particularly true for those people who come into wheelchair use because of a progressive deterioration of mobility as an adult, who may not require or have access to specialist seating services for wheelchair prescription.

To support wheelchair training, initiatives such as the International Society of Wheelchair Professionals (ISWP) (Goldberg et al., 2018) and the World Health Organisation's (WHO) Global Cooperation of Assistive Technology (WHO, 2017) promote collaboration between service providers, policymakers, academics, and wheelchair users. These organisations globally coordinate wheelchair service standards and provide platforms for information exchange. Guidance on skills to be taught and propulsion considerations are offered through the WHO's wheelchair provision guidelines (WHO, 2023) and the Clinical Practice Guidelines for the Preservation of Upper Limb Function following a spinal cord injury (SCI) (Paralyzed Veterans of America Consortium for Spinal Cord, 2005).

Multiple peer‐reviewed MWC training protocols/programs for health professionals to follow are also available, including WheelSeeU (Miller et al., 2019), Roulez Confiance (Rolling with Confidence) (Beaudoin et al., 2021), TEAM Wheels (Giesbrecht et al., 2021), EPIC Wheels (Giesbrecht & Miller, 2017) and the Wheelchair Skills Training Program (WSTP) (Kirby et al., 2022). Innovative training methods such as training apps (Giesbrecht & Miller, 2019; Liu et al., 2019), biofeedback (Rice & Rice, 2017), and virtual reality (Lam et al., 2018) have also been introduced to support training accessibility and confidence in wheelchair use.

Despite their availability, existing wheelchair programs/protocols are not always suitable for the unique physical, cognitive, and affective capacities of diverse wheelchair users because they were developed for specific populations and/or have a tick box approach to their delivery. There is limited guidance around which tailored training approaches, including delivery methods and environmental considerations are more or less likely to work for certain people, in certain situations.

The intent of wheelchair *provision* is well documented—noting a strong emphasis on short‐term outcomes centred largely around wheelchair use, user satisfaction, and activity and participation (Robertson et al., 2023). MWC skill *training* programs are also largely focussed on short‐term outcomes (Tu et al., 2017) with positive changes in wheelchair skills (Keeler et al., 2019), biomechanical efficiency (Chen, 2019; DeGroot et al., 2009) and confidence in MWC use (Beaudoin et al., 2021; Pellichero et al., 2020), Overall, the breadth of intended training outcomes reported on as part of MWC training has not been systematically mapped across the literature.

Considering the global ageing population and the increasing prevalence of chronic and progressive conditions, deeper understanding of tailored training requirements and training programs intentionally designed to consider longer term occupational based outcomes for people coming into MWC use as adults is important for supporting occupational engagement and wellbeing for MWC users and reducing burden on carers and services. This scoping review, therefore, aims to explore the extent, and nature of existing wheelchair training programs and the intended training outcomes for adults coming into MWC use. These findings can identify current knowledge and practice to guide future training approaches.

The WHO International Classification of Functioning (ICF) will be used as a conceptual framework to map/classify the types of intended training outcomes. The WHO International Classification of Functioning (ICF), is an established and internationally recognised framework building on an extensive body of socio‐ecological and biopsychosocial research (Bickenbach et al., 1999). The ICF framework gives structure to the dynamic relationship between outcomes related to person factors, the environmental context, and body structures/functions, including the engagement of wheelchair users' in meaningful occupations. This scoping review explores the following research question and objectives;

What wheelchair training approaches and intended training outcomes are reported for adult manual wheelchair users?

- Objective 1: To document the nature and type of MWC training being delivered to adults commencing wheelchair use.
- Objective 2: To describe which outcomes from MWC training are reported in program evaluations.

## 2 METHODS

### 2.1 Approach to review

A scoping review protocol was developed and prospectively conducted following the Arksey & O'Malley framework (Arksey & O'Malley, 2005). A scoping review was chosen to explore the breadth of relevant literature that exists, including both published and non‐published literature (Arksey & O'Malley, 2005). This approach to the review aimed to capture the emergent and range of wheelchair training approaches and outcomes reported across settings.

### 2.2 Search strategy

The PCC framework (Participants, Concept, Context) was used to guide the review question and selection criteria (Pollock et al., 2021). Initially, a search strategy inclusive of all participant groups trained in MWC use (excluding papers that focussed exclusively on powered wheelchair training or exercise prescription) was undertaken. Reviews, summaries, survey findings, and conference abstracts (where full‐text articles were unable to be located) were also included to ensure that no training approaches were missed and to capture literature addressing MWC training that reflected aetiological and contextual variance. Papers published before 1995 were excluded to capture contemporary training approaches and wheelchair technology, and papers that were not available in English were also excluded.

This initial search strategy was enacted with support from a medical librarian in July 2022 and was updated in September 2023. The databases searched included Cochrane's Library, EMBASE, CINAHL, PubMed, Scopus, EmCare, Medline, ProQuest Nursing, and Allied Health Database. Each database was searched by using MeSH headings (if applicable), synonyms, wildcards, and truncations where appropriate, and all terms are included as Data S1. A search for grey literature was completed in October 2022 and updated in September 2023 using Google Scholar, TROVE, Open Grey NICE, SIGN, ECRI guidelines Trust, TRIP, and focussed websites, including Australian Association of Gerontology and the ISWP website, and using a Google advanced search, including the top 300 results (Haddaway et al., 2015). Reference lists from eligible research papers were screened for further relevant studies. Search results were uploaded and duplicates removed in EndNote and then exported into Covidence Systematic Review software for further deduplication (Haddaway et al., 2015).

### 2.3 Study screening and selection

**FIGURE 1:**
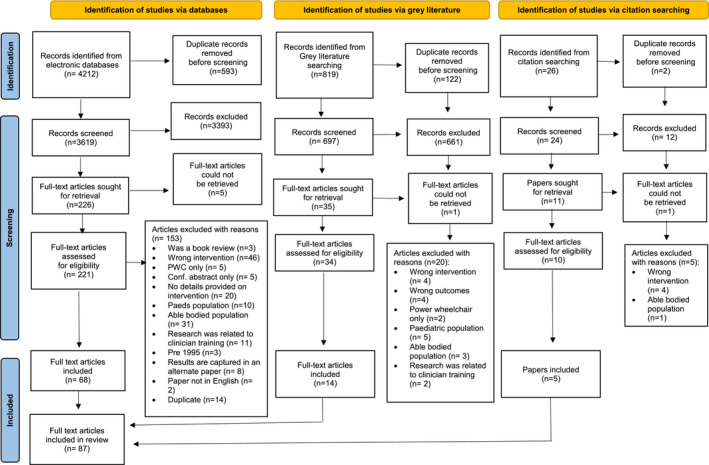
PRISMA‐ScR diagram.

Abstract and full‐text screening were conducted by two independent reviewers for 4212 articles. Because of the size and broad scope of literature found at the stage of full‐text screening, the PCC framework and inclusion/exclusion criteria were iteratively refined to focus on adult wheelchair users with chronic or progressive conditions who may come into wheelchair use later in life, hence excluding able‐bodied persons simulating wheelchair users, clinicians, students and also paediatric MWC users. Reviewers discussed conflicts, until consensus was achieved. Following screening, 87 articles were included in the review, as shown in the PRISMA‐ScR diagram in Figure 1.

### 2.4 Data charting and synthesis

Three types of data were included in this review: (1) study characteristics and characteristics of training programs, (2) outcomes reported across quantitative papers, and (3) discussions related to training being delivered and outcomes reported in qualitative/other literature. The ICF was used as a deductive framework to synthesise data types two and three. This mapping was completed interpretively by the primary reviewer, but the research team collectively refined, categorised, and described data under the broad ICF components.

For the first two data types, the research team agreed on extraction criteria in accordance with the research question. Two reviewers independently piloted the criteria across 10 studies. The criteria were adjusted in response to the pilot, and the data charting table was updated as analysis progressed. Study characteristics and characteristics of training programs were extracted, including year of publication, country of origin, type of paper, participant demographics (reason for wheelchair use, amount of wheelchair experience, age, sex), training context (location of training, group vs. individual approach, provider of training) and training structure (how many training sessions, duration of training sessions, time period over which training was provided, total amount of training). The second data type extracted from quantitative papers included outcomes reported, outcome tools, and timing of recording outcomes.

**FIGURE 2:**
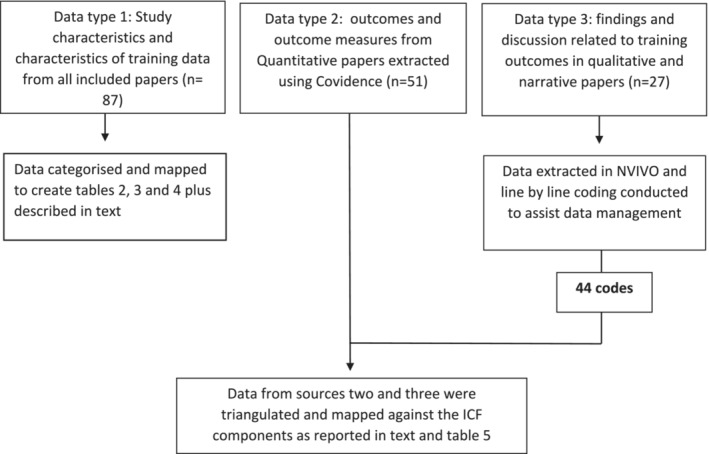
Data analysis procedure.

The third data type that related to the review question was training outcomes as reported in the findings and discussion sections of papers with qualitative and narrative data (e.g., qualitative research, scoping reviews, literature reviews, and systematic reviews). Papers were imported into NVIVO for coding and management of the data. Line‐by‐line coding of qualitative and narrative data in NVIVO generated 44 initial codes. Memo and annotation tools were used to keep an audit trail of decisions made and to facilitate discussion and consensus amongst reviewers to ensure data triangulation. Figure 2 provides an outline of the data synthesis process.

### 2.5 Positionality statement

All members of the review team are of allied health background, including two speech pathologists, one physiotherapist, one podiatrist, and three occupational therapists, all who have published or have clinical experience in the area of assistive technology. The primary author (KC) has worked clinically in the area of wheelchair provision and MWC training with adults. Four authors were involved in conceiving and designing the research review and extracting and analysing data. All authors contributed to the screening of articles and the revision of the manuscript.

## 3 RESULTS

### 3.1 Study characteristics

All 87 included papers were published between 2001 and 2023, most frequently in 2015 (*n* = 13), then 2021 (*n* = 9), 2022 (*n* = 8), 2013 (*n* = 6) and 2019 (*n* = 6). Papers were published in 18 countries, mostly arising from North America (United States = 27 and Canada = 29). Papers included both original research papers (*n* = 59) that were methodologically diverse and other papers, including systematic/scoping and literature reviews (*n* = 10), training protocols (*n* = 9), and summaries and opinion/position papers (*n* = 7). Table 1 details the type of papers included, with the aims of the papers presented in Data S2. Forty‐seven papers had data relating to quantitative outcome measures and 27 qualitative/other literature included discussion related to training being delivered or outcomes reported.

**TABLE 1:** Type of paper.

Type of paper	Number	Author
Randomised control trial	25	Best et al., 2005^b^; Best et al., 2016, ^b^; Bonaparte et al., 2004, ^b^; Chen, 2019, ^b^; Giesbrecht & Miller, 2019, ^b^; Giesbrecht et al., 2017, ^b^; Kirby et al., 2001, ^b^; Kirby et al., 2008^b^; Kirby et al., 2016, ^b^; Limroongreungrat et al., 2009 ^b^; MacPhee et al., 2004, ^b^; McClure et al., 2010, ^b^; Miller et al., 2019, ^b^; Öztürk & Ucsular, 2011, ^b^; Park & Jung, 2022, ^b^; Rice et al., 2013, ^b^; Rice et al., 2014, ^b^; Rice et al., 2015, ^b^; Routhier et al., 2012, ^b^; Smith et al., 2009, ^b^; Van Der Scheer et al., 2015, ^b^; Van Der Scheer et al., 2016, ^b^; Worobey et al., 2016, ^b^; Yeo & Kwon, 2018, ^b^; Wang et al., 2015, ^b^
Systematic/scoping/literature review^c^	10	Caro & da Cruz, 2020, ^a^; Keeler et al., 2019, ^a^; Kehrer et al., 2021, ^a^; Kirby et al., 2006, ^a^; Lam et al., 2018, ^a^; Abdul Rahim et al., 2021, ^a^; Rice & Rice, 2017^a^; Symonds et al., 2018, ^a^; Tu et al., 2017, ^a^; Zwinkels et al., 2014, ^a^
Training program^c^	9	Denison, 2013; Northwestern Regional Spinal Cord Injury System, 2021; Physiopedia, 2022; Push Mobility, 2023; Robinson & Savary, 2022; The Back up Trust, 2022; WHO, 2012
Pre‐design and post‐design	9	Desai et al., 2013, ^b^; Furmaniuk et al., 2010, ^b^; Giesbrecht, Miller, & Woodgate, 2015b; Kirby et al., 2004, ^b^; Morgan, Tucker, et al., 2017, ^b^; Quiñones‐Uriostegui et al., 2023, ^b^; Richter et al., 2011, ^b^; Rodgers et al., 2001, ^b^; Tasiemski et al., 2021, ^b^
Qualitative research	9	Chaar & Archambault, 2022, ^a^; Giesbrecht et al., 2014, ^a^; Giesbrecht, Miller, & Woodgate, 2015, ^a^; Liu et al., 2019, ^a^; Morgan, Engsberg, & Gray, 2017, ^a^; Pellichero, 2020, ^a^; Pouvrasseau et al., 2017; Rusek et al., 2021, ^a^; Standal & Jespersen, 2008, ^a^
Summary/text opinion/position paper^c^	7	Budai & Murdoch, 2019, ^a^; Cooper et al., 2005, ^a^; Genova et al., 2021, ^a^; Giesbrecht, Best, & Miller, 2015^a^; Michael, 2013, ^a^; Requejo et al., 2008, ^a^; Sawatzky et al., 2015, ^a^
Survey	5	Best et al., 2015, ^b^; Kirby et al., 2015, ^b^; Kirby et al., 2020, ^b^; Kirby et al., 2021, ^b^; Mathis & Gowran, 2021, ^b^
Cohort study	4	Blouin et al., 2015, ^b^; DeGroot et al., 2009, ^b^; Kotajarvi et al., 2006, ^b^; Taylor et al., 2014, ^b^
Protocol	2	Srinivasan 2023, ^b^; Giesbrecht et al., 2021, ^b^
Mixed methods	2	Beaudoin et al., 2021, ^a^ ^,^ ^b^; Charlton et al., 2021, ^a^ ^,^ ^b^
Cross sectional	2	Charbonneau et al., 2013, ^b^; Choi et al., 2020^b^
Case study	2	Garrett et al., 2011, ^b^; Rice et al., 2010, ^b^
Participatory action research	1	Arlati et al., 2019, ^a^

^a^ Qualitative/narrative papers where data about training delivery and outcomes reported have been extracted.

^b^ Papers where outcomes reported in quantitative papers and quantitative tools used have been extracted.

^c^ Not research.

### 3.2 Characteristics of study participants

The wheelchair users who participated in the MWC training programs (*n* = 47) reported in original research papers are explained in Table 2. Across all studies, there were 2413 participants, with three times more men (*n* = 1822; 75%) than women (*n* = 508; 21%) (3% *n* = 83 unspecified sex). MWC users were predominantly participants with an SCI (*n* = 2020; 84% of total participants) and were an average age of 45.8 ± 11.56 years, with only four papers having an average user age over 65 years (Giesbrecht & Miller, 2017; Giesbrecht, Miller, Jin, et al., 2015; Miller et al., 2019; Park & Jung, 2022). At the time of wheelchair training, MWC users had a wide range of wheelchair experience (3 weeks to 30 years of wheelchair use). Papers commonly focussed on participants with SCI (*n* = 21 papers) or mixed user groups (*n* = 20 papers) (i.e., stroke, SCI, MS, amputees, and able‐bodied persons). A small number of studies focussed on specific conditions other than SCI, including stroke (*n* = 2) (Charbonneau et al., 2013; Park & Jung, 2022), dementia (*n* = 1) (Smith et al., 2009), multiple sclerosis (MS) (*n* = 1) (Rice et al., 2015) and amputees (*n* = 1) (Charlton et al., 2021).

**TABLE 2:** Participant characteristics.

Author	No. of participants	Specific diagnosis							Diagnosis not specified	Other conditions	Average age (years)	Gender	Years of experience in MWC use (average)
		MS	SCI	Amputee	OA	CP	Polio	Stroke					
Beaudoin et al., 2021	19	3	3			2		2		2‐ neuromuscular disease 7‐ spina bifida	45	9 M, 10 F	14
Best et al., 2005	20		2	10				5		3‐ peripheral neurological disorder	49	15 M, 5 F	0.91
Best et al., 2016	28	2	19			4			3		48.7	22 M, 6 F	13.6
Blouin et al., 2015	18		18								42.4	16 M, 2 F	14.8
Bonaparte et al., 2004	22		2							10 MSK 10 able‐bodied persons	34.7	13 M, 9 F	>2 years
Chaar & Archambault, 2022	6								6		WC user56 WC trainer33.8	WC user: 5 M, 1 F WC trainer: 4 M, 1 F	>3 years
Charbonneau et al., 2013	18							18			60	13 M, 5 F	.25 years
Charlton et al., 2021	11			11							58.7	9 M, 2 F	0.05
Chen, 2019	8	2	5			1					47.5	7 M, 1 F	16
Choi et al., 2020	4		4								47.2	2 M, 2 F	.6 year
DeGroot et al., 2009	9	1	6			1				1‐ spinal muscular atrophy	36.8	6 M, 3 F	10
Desai et al., 2013	13		13								NS	NS	NS
Furmaniuk et al., 2010	40		40								29.4	40 M	>5 years
Garrett et al., 2011	2		2								NS	NS	NS
Giesbrecht, Miller, & Woodgate, 2015	2		1	1							66	2 M	20.2
Giesbrecht, Best, & Miller, 2015	10								10		55–85	7 M, 3 F	4–60 years
Giesbrecht et al., 2017	18								18		66.1	13 M, 5 F	<2 years
Giesbrecht & Miller, 2019	18	1	2	3	3			1		2‐ Parkinson's 5‐ ortho injury 1 Arteriovenous malformation	65	13 M, 5 F	1.7
Kirby et al., 2016	106		106								39	101 M, 5 F	17.4
Kirby et al., 2001	42		6	4					2	30‐ able‐bodied persons	30	10 M, 32 F	NS
Kirby et al., 2008	30								15	15‐able bodied persons	37.5	NS	NS
Kotajarvi et al., 2006	18		18								38.4	16 M, 2 F	14
Limroongreungrat et al., 2009	28		28								33.9	NS	NS
MacPhee et al., 2004	35									20‐ MSK 15‐ Neuro	59	26 M, 9 F	.19
Miller et al., 2019	18		2	3					13		66.2	7 M, 11 F	4.3
Morgan, Tucker, et al., 2017	6		6								38	4 M, 2 F	1.27
McClure et al., 2010	31		31								41.4	21 M, 10 F	NS
Öztürk & Ucsular, 2011	24		13	2	1	1	1	1		1‐ THR 1‐ Meningitis 3‐ hip dislocation	33	11 M, 13 F	8.85
Park & Jung, 2022	24							24			68.4	14 M, 10 F	2
Pellichero 2020	11	3	2	2	2		2				64.6	3 M, 8 F	4.8
Quiñones‐Uriostegui et al., 2023	12		12								35.3	10 M, 2 F	5.6
Rice et al., 2010	1		1								45.6	1 M	11
Rice et al., 2015	14	14									53.85	4 M, 10 F	<17
Rice et al., 2013	27		27								40.4	24 M, 3 F	17.9
Rice et al., 2014	37		37								37	28 M, 9 F	NS
Richter et al., 2011	31		23			1				6‐ spina bifida 1‐ spinal lipoma	34.1	27 M, 4 F	15
Rodgers et al., 2001	19		15							2‐ multi‐trauma 1‐ bilateral tarsal tunnel syndrome	54.4	16 M, 3 F	17
Routhier et al., 2012	39	8	19	9					3		46	27 M, 12 F	.29
Smith et al., 2009	13									13‐ dementia	NS	NS	NS
Standal & Jespersen, 2008	20		11							9‐ unspecified neurological	43	16 M, 4 F	.057–30
Tasiemski et al., 2021	19		4			3	5		7		33	13 M, 6 F	24
Taylor et al., 2014	1,376		1,376								38	1,114 M, 262 F	.08
Van Der Scheer et al., 2015	16		16								54	11 M, 5 F	>10 years
Van Der Scheer et al., 2016	29		29								56	22 M, 7 F	18
Worobey et al., 2016	79		79								40.5	69 M, 10 F	13.2
Yeo & Kwon, 2018	24		24								35	19 M, 5 F	2.85
Wang et al., 2015	18		18								34	12 M, 6 F	0 years
TOTAL	2,413	34	2020	45	6	13	8	51	77	159	45.8	1822 M, 508F, 83NS	9.6

Abbreviations: CP, cerebral palsy; MS, multiple sclerosis; NS, not specified; OA, osteoarthritis; SCI, spinal cord Injury; F, Female; M, Male.

### 3.3 Wheelchair training structure

As depicted in Table 3, the structure of MWC training (number, duration, length of sessions) was not consistent across original research papers (*n* = 47). The number of wheelchair training sessions delivered in studies ranged from single sessions (*n* = 4) to 104 sessions (Furmaniuk et al., 2010), and their length varied from 10 minutes (Kotajarvi et al., 2006) to 300 minutes (Standal & Jespersen, 2008). Some wheelchair training programs consisted of 1 day (*n* = 3) with one being as many as 730 days (Furmaniuk et al., 2010).

**TABLE 3:** Number, duration, and time period that wheelchair training was provided.

Authors name	How many training sessions	Duration of each training session (minutes)	Time period over which training was provided (days)	Total training provided (hours)
Beaudoin et al., 2021	6	120	21–42	12
Best et al., 2005	3 to 5	60	69	4.5
Best et al., 2016	6	90	21–42	9
Blouin et al., 2015	1	25	1	0.46
Bonaparte et al., 2004	RBS group: 2–9 PBS + RBS group: 2–9	RBS group: 30 RBS + PBS group: 30	RBS group: 21 RBS + PBS group: 21	RBS group: .71 RBS + PBS group: .75
Chaar & Archambault, 2022	1	30	30	0.5
Charbonneau et al., 2013	1	Not reported	1	Not reported
Charlton et al., 2021	2.50	45	21	2.25 hours
Chen, 2019	Repetitive training and education group = 7 Education only = 1	Education = 30 Repetitive training = 60	21	Repetitive training and education group = 6.5 hours Education only = .5 hours
Choi et al., 2020	10	30	Not reported	5
DeGroot et al., 2009	5	20–40	50–70	1.66–3.33
Desai et al., 2013	>8	120	8	24
Furmaniuk et al., 2010	104	120	730	208
Garrett et al., 2011	Insufficient detail			
Giesbrecht & Miller, 2019	2 in person Online facilitated 4 unstructured use	In person: 1 × 120, 1 × 60 Unstructured use: 75–150 Online: Unspecified	28	In person: 3 mHealth online: 5–10 Unstructured WC use: 5 Total = 13–18
Giesbrecht, Miller, & Woodgate, 2015b	2× in person training session 16–20 of home practice	In person: 1 × 120 and 1 × 60 Home practice = 15–30	28	In person training: 3 At home training practice: 4–10
Giesbrecht et al., 2017	2 in person Online facilitated Unstructured	In person: 1 × 60, 1 × 120 75–150 mHealth program: 75–150 Unstructured WC use: 75	28	In person: 3 mHealth: 5–10 Unstructured WC use: 5 Total = 13–18
Giesbrecht et al., 2021	8 to 24	15–30	28	8 to 48
Kirby et al., 2001	Unclear	20	Unclear	Unclear
Kirby et al., 2004	1	50	1	0.83
Kirby et al., 2008	5	37	5	3
Kirby et al., 2016	5	30–45	49–93	2.5 to 3.75
Kotajarvi et al., 2006	4	10	2	0.66
Limroongreungrat et al., 2009	1	30	Not reported	1
MacPhee et al., 2004	6	30	25	3
McClure et al., 2010	Not reported	Not reported	Not reported	Not reported
Miller et al., 2019	6	90		
Morgan, Tucker, et al., 2017	9	90	21–30	13.5
Öztürk & Ucsular, 2011	12	45	28	9
Park & Jung, 2022	Unclear	Unclear	6 weeks	Unclear
Pellichero, 2020	6	90		9
Quiñones‐Uriostegui et al., 2023	Not reported	Not reported	Not reported	Not reported
Rice et al., 2010	4	Not reported	Not reported	Not reported
Rice et al., 2013	Feedback group: 4 + weekly phone calls Instruction‐only group = 4 + weekly phone calls	Not reported	20	Unclear
Rice et al., 2014	Unclear	180–240	Not reported	Unclear
Rice et al., 2015	1 face‐to‐face training session + phone calls	Not reported	90	Unclear
Richter et al., 2011	Not reported	Not reported	Not reported	Not reported
Rodgers et al., 2001	18	Unclear	42	Unclear
Routhier et al., 2012	4–8 Average 5.9	45–60	27	5.36
Smith et al., 2009	8	Not reported	28	Unclear
Standal & Jespersen, 2008	14 to 15	180–300	17	42–75
Tasiemski et al., 2021	9	180–240	6	29
Van Der Scheer et al., 2015	32	18 or 24 minutes for the first 4 sessions 30 minutes thereafter.	112–126	15.4
Van Der Scheer et al., 2016	32	18 or 24 minutes in the first 4 sessions 30 minutes for sessions after this	112–126	15.4
Worobey et al., 2016	5	90	42	7.5
Yeo & Kwon, 2018	24	60	56	24
Wang et al., 2015	6 to 8	30	21–28	3 to 4

MWC training was commonly facilitated by allied health professionals, including physiotherapists (*n* = 14), occupational therapists (*n* = 13), allied health assistants (*n* = 4), exercise physiologists (*n* = 2), kinesiologists (*n* = 1) or peer wheelchair users (*n* = 8). Table 4 provides information about who provided training, where the training was located and how it was structured across original papers. Support for wheelchair use was also achieved through involving carers and/or friends in wheelchair training or via small group training (two to three people) (*n* = 3) or large (over four people) group training (*n* = 6). Training was also facilitated/proposed to be facilitated by technology, including tablet/computer‐based online learning (*n* = 5), virtual reality technology (*n* = 5), rehabilitation technology (i.e., simulators, treadmills, and instrumented wheels) (*n* = 7) and provision of real‐time visual feedback.

**TABLE 4:** Provider, location, and mode of training.

Authors name	Provider of wheelchair training					Location training took place				Group/individual training		
	PT	OT	AHA	Peer WC user	Other/NS	Inpatient setting	Community	Online	Other/NS	Individual	Small group	Large group
Beaudoin et al., 2021				X			X					X
Best et al., 2005					Kinesiologist		X			X		
Best et al., 2016				X			X				X	
Blouin et al., 2015				NS					Sports training lab	X		
Bonaparte et al., 2004				NS					Kinesiology lab			
Chaar & Archambault, 2022				NS		X				X		
Charbonneau et al., 2013				NS		X				X		
Charlton et al., 2021		X	X			X					X	
Chen, 2019				NS			X		Sports training lab	X		
Choi et al., 2020	X					X				X		
Srinivasan, 2023				NS			X	X		X		
DeGroot et al., 2009				NS					Sports training lab	X		
Desai et al., 2013				NS			X			NS		
Furmaniuk et al., 2010	X				EP		X					X
Garrett et al., 2011					NS	X				X		
Giesbrecht & Miller, 2019		X				X	X	X		X		
Giesbrecht, Miller, & Woodgate, 2015b					NS		X	X		X		
Giesbrecht et al., 2017		X				X	X	X		X		
Giesbrecht et al., 2021				X			X	X		X		
Kirby et al., 2001	X								NS	X		
Kirby et al., 2004					NS		X			X		
Kirby et al., 2008					NS	X				X		
Kirby et al., 2016	X	X	X				X			X		
Kotajarvi et al., 2006					NS				Sports training lab	X		
Limroongreungrat et al., 2009					NS				NS	NS		
Liu et al., 2019					NS			X		X		
MacPhee et al., 2004	X	X				X				X		
McClure et al., 2010	X	X				X				X		
Miller et al., 2019	X	X		X		X					X	
Morgan, Tucker, et al., 2017		X					X			X		
Öztürk & Ucsular, 2011	X					X				X		
Park & Jung, 2022				NS					NS			X
Pellichero et al., 2020				X		X						
Quiñones‐Uriostegui et al., 2023					NS				NS	X		
Rice et al., 2010				NS				NS		X		
Rice et al., 2013				NS					Sports training lab	X		
Rice et al., 2014	X	X				X	X			X		
Rice et al., 2015	X	X				X			University lab	X		
Richter et al., 2011				NS					Sports training lab	X		
Rodgers et al., 2001				NS					Sports training lab			
Routhier et al., 2012		X				X				X		
Smith et al., 2009					NS				Residential care facility			
Standal & Jespersen, 2008	X	X		X	EP	X						X
Tasiemski et al., 2021				X					Active rehab training camp			X
Taylor et al., 2014	X	X				X				NS		
Van Der Scheer et al., 2015			X			X				X		
Van Der Scheer et al., 2016			X			X				X		
Worobey et al., 2016				NS			X					X
Yeo & Kwon, 2018	X								NS	X		
Wang et al., 2015	X			X		X				X		
Total	14	13	4	8	23‐ NS 2‐ EP 1‐Kinesiologist	21	16	6	9‐ lab setting	34	3	6

Abbreviations: AHA, allied health assistant; EP, exercise physiologist; NS, not specified; OT, occupational therapist; PT, physiotherapist; WC, wheelchair.

Training mostly occurred across inpatient and rehabilitation settings (*n* = 21), followed by home/community (*n* = 16), online (*n* = 6), sports training/kinesiology laboratories (*n* = 9), training camp (*n* = 1) and a residential care facility (*n* = 1). Several papers emphasised the importance of implementing training across multiple environments and terrains; however, many wheelchair users receive little to no specific training within community settings (Kirby et al., 2020; Kirby et al., 2021; Mathis & Gowran, 2021).

The focus of MWC training was diverse, including education on exercise/strength and endurance, overuse injury and safe biomechanical propulsion (*n* = 13), instruction and practice of individual or multiple wheelchair skills (i.e., going forwards, going up slight inclines, and wheelies) (*n* = 30) or goal‐based training (*n* = 12). Fifty‐nine of the 87 included papers identified a wheelchair training program/protocol, the most prevalent being the WSTP (*n* = 9) or Adapted WSTP (*n* = 6). However, wheelchair trainers recognised that wheelchair training is often ad hoc and varied in its delivery (Best et al., 2015; Kirby et al., 2020; Kirby et al., 2021; Mathis & Gowran, 2021).

### 3.4 Reporting of intended wheelchair training outcomes

Findings about intended MWC training outcomes were derived from the synthesis of both quantitative and qualitative data. Figure 2 details this synthesis process. The intended outcomes and methods used to capture these outcomes are explained in Table 5. The majority of included papers captured outcomes immediately following delivery of MWC training or within four weeks post‐training. However, five papers looked at outcomes 30–90 days after training was completed (Beaudoin et al., 2021; Desai et al., 2013; Rice et al., 2013; Routhier et al., 2012; Yeo & Kwon, 2018), four at six months after training (Giesbrecht et al., 2021; Kirby et al., 2004; McClure, 2010; Miller et al., 2019) and three at 12 months after training (Kirby et al., 2016; Quiñones‐Uriostegui et al., 2023; Rice et al., 2014).

**TABLE 5:** Intended WC training outcomes mapped against the ICF.

	Intended training outcomes	Methods used	Paper names
Body structures and functions (n = 18) 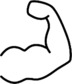	Biomechanical outcomes (*n* = 13)(includes stroke frequency, peak, and average force, contact angle, Clinical practice guideline adherence, push loop/length, and velocity)	Video motion capture, SMART wheel,^a^ ergometer	Blouin et al., 2015; Chen, 2019, DeGroot et al., 2009; Kotajarvi et al., 2006; Limroongreungrat et al., 2009; McClure et al., 2010; Morgan, Tucker, et al., 2017; Rice et al., 2010; Rice et al., 2013; Rice et al., 2014; Rice et al., 2015; Richter et al., 2011; Van Der Scheer et al., 2015
	Fitness (*n* = 3) UL strength, energy expenditure, and aerobic fitness	Dynamometer/MMT, Van Lieshout test (VLT‐SV)METS, METS	Rice et al., 2015; Van Der Scheer et al., 2016; Yeo & Kwon, 2018
	Pain (*n* = 2)	Wheelchair Users Shoulder Pain Index (WUSPI), Pain Rating Scale	McClure et al., 2010; Rice et al., 2014
Activities and participation (n = 39) 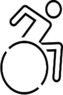	Wheelchair skills (n = 29)includes broad or specific wheelchair skills including capacity, performance and safety	WST‐Q, WST, the Queensland evaluation wheelchair skills, wheelie competence test, VAS, observational counting	Beaudoin et al., 2021; Best et al., 2005; Best et al., 2016; Bonaparte et al., 2004; Charlton et al., 2021; Choi et al., 2020; Srinivasan 2023; Desai et al., 2013; Furmaniuk et al., 2010; Garrett et al., 2011; Giesbrecht & Miller, 2019; Giesbrecht, Miller, & Woodgate, 2015; Giesbrecht et al., 2017; Giesbrecht et al., 2021; Kirby et al., 2004; Kirby et al., 2008; Kirby et al., 2016; MacPhee et al., 2004; Miller et al., 2019; Morgan, Tucker, et al., 2017; Öztürk & Ucsular, 2011; Park & Jung, 2022; Quiñones‐Uriostegui et al., 2023; Routhier et al., 2012; Tasiemski et al., 2021; Van Der Scheer et al., 2016; Worobey et al., 2016; Yeo & Kwon, 2018; Wang et al., 2015
	Functional mobility (*n* = 3)	FIM Mobility Scores, interviews	Best et al., 2016; Charlton et al., 2021; Desai et al., 2013
	Community participation (*n* = 4)	Korean version of modified Barthel Index Life Space Assessment, Actigraph datalogger, Late Life Function and Disability Index	Giesbrecht & Miller, 2019; Giesbrecht et al., 2017; Giesbrecht et al., 2021; Park & Jung, 2022
	ADL/goal achievement (*n* = 3)	GAS, COPM	Charlton et al., 2021; Miller et al., 2019; Park & Jung, 2022
Personal factors (n = 27) 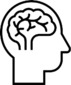	Wheelchair self‐efficacy/confidence (*n* = 13)	WheelConM‐2, interviews	Beaudoin et al., 2021; Best et al., 2005; Best et al., 2016; Bonaparte et al., 2004; Charlton et al., 2021; Choi et al., 2020; Giesbrecht & Miller, 2019; Giesbrecht, Miller, & Woodgate, 2015; Giesbrecht et al., 2017; Giesbrecht et al., 2021; Kirby et al., 2008; Miller et al., 2019; Pellichero et al., 2020
	Health‐related quality of life (*n* = 7)	Satisfaction with Life Scale, Health‐Related Quality of Life Index, Short Form 36 Health Survey Questionnaire, PIADS, Craig Handicap Assessment and Reporting Technique	Beaudoin et al., 2021; Giesbrecht & Miller, 2019; Giesbrecht et al., 2017; Giesbrecht et al., 2021; MacPhee et al., 2004; McClure et al., 2010; Rice et al., 2014
	Satisfaction with WC performance (*n* = 7)	WhOM‐2	Beaudoin et al., 2021; Best et al., 2016; Desai et al., 2013; Giesbrecht, Miller, & Woodgate, 2015b; Giesbrecht et al., 2021; Kirby et al., 2008; Pellichero et al., 2020
Environmental factors (n = 3) 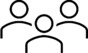	Social environments and relationships (*n* = 3)	Interviews	Beaudoin et al., 2021; Pellichero et al., 2020; Quiñones‐Uriostegui et al., 2023

Abbreviations: COPM, Canadian occupational performance measure; FIM, functional independence measure; GAS, goal attainment scale; MMT, manual muscle testing; METS, metabolic equivalent of task; PIADS, Psychosocial Impact of Assistive devices Scale; UL, upper limb; VAS, visual analogue scale; WheelConM, wheelchair use confidence scale; WST, wheelchair skills test; WST‐Q, wheelchair skills test questionnaire; WhOM, the wheelchair outcome measure.

^a^ Smart‐Wheel is a specialised wheelchair wheel that measures propulsion parameters, including forces applied to the pushrim, push length, push frequency, and velocity during wheeling.

Intended training outcomes were mapped across broad ICF components, namely, *activity and participation*, *personal factors*, *body structures and functions*, and *environmental factors*, and were reported based on prominence within the reviewed literature.

#### 3.4.1 Activity and participation

ICF‐reported outcomes were most frequently reported (*n* = 39) and related to the execution of a task or action by an individual through involvement in daily life. Papers predominantly focussed on outcomes related to the acquisition of one or more specific wheelchair skills (i.e., going up a kerb, rolling forwards, and descending a ramp) (*n* = 29). Papers focussed less on outcomes for community participation (*n* = 4) and activities of daily living and occupational participation/goal acquisition (*n* = 3). These participation‐focussed papers reported outcomes, including functional wheelchair training such as toileting, dressing and bathing within home‐like environments, carrying objects, negotiating shopping centres, getting through doorways, and navigating everyday activities and functional mobility.

#### 3.4.2 Personal factors

ICF‐reported outcomes were present in 27 papers. These were psychological and coping styles that were not part of a health condition but contributed to experiences of health and how people interacted with their environment. Reported outcomes included self‐efficacy/confidence (*n* = 13), satisfaction with wheelchair performance (*n* = 7), and health‐related quality of life (*n* = 7).

#### 3.4.3 Body structures and functions

ICF‐reported outcomes were present in 18 papers and related to anatomical parts of the body and their physiological function, including propulsion biomechanics (*n* = 13), fitness (*n* = 3), and pain (*n* = 2). Many of the biomechanical outcomes were measured using technology, including video motion capture, wheelchair ergometers and dynamometers, and Smart Wheels.

#### 3.4.4 Environmental factors

ICF‐reported outcomes only arose in three papers and included the physical, social, and attitudinal environment in which people conducted their lives. Reported outcomes focussed on the social environments where wheelchair training was delivered, including outcomes related to a sense of belonging due to social relationships.

## 4 DISCUSSION

The purpose of this scoping review was to report the range and nature of existing MWC training approaches for adults who are new to wheelchair use and the intended outcomes of the training. Data were organised and analysed to describe MWC training approaches, and the ICF was used as a deductive framework to map intended training outcomes as reported in the literature.

Existing wheelchair training literature for adult wheelchair users predominantly targets wheelchair users with SCI and younger demographic groups. Adults with progressive or deteriorating conditions are not well represented within the MWC training literature and papers frequently excluded those with reduced cognition from MWC training programs (Miller et al., 2019; Park & Jung, 2022). Additionally, in many circumstances, papers used able‐bodied persons to mimic wheelchair users (Leving et al., 2015; Sakakibara et al., 2013; Yao et al., 2012), which is not representative of the physical and psychological status of wheelchair users. Wheelchair training approaches reported within these papers therefore may not be applicable to adults who come into wheelchair use because of progressive and deteriorating health conditions and may require different psychosocial and physical considerations when implementing MWC training. This makes it difficult for occupational therapists to choose and justify the most appropriate approach to training for people who are adults and need to use a wheelchair for reasons other than SCI.

The included papers lacked consistency regarding the program design of the wheelchair training, including the frequency and length of training. This appeared to be irrespective of the content being taught, the demographic target group, and the location of training delivery. It was unclear whether these training structures were developed pragmatically, that is, considering factors such as costs and resources of training, or whether training structure was developed with purpose. Further investigation into different lengths and frequencies of training to understand what works best for particular demographic groups is required. This information is particularly important given that personal factors such as fatigue, attention, and motivation for people with chronic and progressive conditions may influence participation in training and the time required to develop and retain skills and then transfer these skills into their everyday life. Without such evidence, it is difficult to make policy and funding arguments for providing tailored wheelchair training.

Findings from this review suggest that wheelchair training is being delivered by a diverse range of trainers, including clinicians and peer MWC trainers. Peer‐based training has become more prevalent (Webel et al., 2010) on the premise that peers often share a common culture and ability to share knowledge about problems/challenges experienced. In the context of wheelchair training, peer involvement has also been used to create belonging, provide opportunity to share ideas/solve practical challenges, and allow for observational learning (Beaudoin et al., 2021; Pellichero et al., 2020; Standal & Jespersen, 2008). Based on this, programs have been developed such as the Wheelchair Skills College (Wheelchair Skills College, 2024) and the Back Up Trust (Back up Trust, 2022) based in the United Kingdom and the Skills for Independence in Australia (AQA, 2024), which are not included in our review because of the recency of their development. Also, currently, evidence for these programs is anecdotal, requiring a rigorous evaluation of outcomes.

Research exploring clinician‐led training has found that they may lack time/resources, have a perceived lack of understanding of difficulties associated with wheelchair use (Standal & Jespersen, 2008), and may be considered too risk‐averse (Caro & da Cruz, 2020; Pellichero et al., 2020; Rusek et al., 2021). Conversely, clinician trainers are perceived to play an important role in supporting confidence and self‐efficacy through facilitating problem solving, providing information, and working collaboratively with clients to address anxiety/fear towards wheelchair use (Giesbrecht, Miller, & Woodgate, 2015b; Pellichero et al., 2020). Although research comparing clinician and peer‐led MWC training is required, qualitative findings suggest that peer MWC trainers and clinicians could work together to provide training (Pellichero et al., 2020).

The majority of programs included in this review were either within community or laboratory/indoor settings and did not move between settings. They also frequently used prescriptive protocols within manufactured training environments. This prescriptive approach may have been intentional, in an effort to create consistent and safe training environments to build confidence. Considering that the average length of hospital stay within Australia is decreasing (Reid et al., 2023), MWC training in the community or online training may be indicated, particularly for adults with progressive and deteriorating conditions that may not require extended hospital stay to learn how to use a MWC.

Online training or virtual reality to support training may also increase accessibility for people living rurally and remotely (Giesbrecht, Miller, Jin, et al., 2015; Liu et al., 2019), reduce staffing costs associated with training, and provide a motivating, risk‐free environment to build confidence and skill progression (Symonds et al., 2018). Although this approach may increase the diversity of available MWC training programs, this technology is not readily accessible to all wheelchair service providers, particularly within lower/middle‐income countries. Training situated in environments where MWC users will be using their wheelchairs is valued (Morgan, Engsberg, & Gray, 2017) and may facilitate improvements in occupational performance outcomes. Community‐based training remains a logical step towards contextualising wheelchair use to the environment of the user.

Intended outcomes of the training programs were mapped to all the ICF components; they focussed particularly on instruction for individual wheelchair skill attainment (activities and participation), building confidence in wheelchair use (personal factors), and efficient propulsion biomechanics (body structures and functions). Although it can be inferred that developing propulsion skills will translate into reduced upper limb pain/more efficient wheelchair use, or that increased confidence and individual wheelchair skills attained can be integrated to support engagement in occupational performance, this is only going to occur if the specific movements are applied to individual circumstances. Prescriptive protocols for developing wheelchair skills, by their very nature, do not foreground individual wheelchair users' goals and priorities, and this may not prepare them for longer term wheelchair use in complex environments nor consider their ability to partake in meaningful activities and occupations. Given a key underlying reason for MWC training is to support engagement in the community and to enable participation in desired occupations, future MWC training programs need to evaluate longer term participatory outcomes (Gowran et al., 2022). Without this information, it is difficult for occupational therapists to draw definitive recommendations about MWC training approaches that will support user occupational performance and community participation.

### 4.1 Conclusion

Wheelchair training is an important part of occupation‐based practice as it enables users to participate in their chosen occupations, supporting their health and wellness. This review identifies the need for a tailored approach to MWC training that addresses the individual needs of wheelchair users and their context. Current wheelchair training approaches focus largely on practicing skills, building confidence, and educating on efficient propulsion rather than on participation in occupations and/or environmental circumstances in which wheelchairs are used. Future MWC training designs need to consider transition across different healthcare settings and be goal based to ensure that outcomes of training are occupationally based and focussed on longer term outcomes.

### 4.2 Limitations

As papers on wheelchair training approaches were limited to English only, wheelchair training programs included in this review may be designed for Western contexts. This review did not include papers with able‐bodied persons simulating wheelchair users, clinicians/students being trained or those under the age of 18; therefore, some training approaches were not captured in this review. Additionally, although this review did consider grey literature, health, and community service organisations have likely developed their own wheelchair training protocols that are not published or available through online sources and these approaches have therefore not been captured; this includes the emerging peer‐led MWC training programs within Australia.

### 4.3 Implications for practice and future research

Current wheelchair provision guidelines provide little insight into the environmental context and circumstances for the provision of MWC training, instead focussing on prescriptive processes that are mostly developed and evaluated with specific populations (WHO, 2023). Through using occupation‐based approaches to equip wheelchair users with effective skills, occupational therapists support the person's engagement in their chosen occupations. Fundamentally, MWC training programs that have a stronger focus on occupational engagement and longer‐term participatory outcomes are required to ensure that skills learned are being translated into everyday life. Future research is required to explore longer term implications of wheelchair training, including the incidence of upper limb injury and the efficacy of tailored wheelchair training in individual environmental contexts for diverse groups of people, including those with progressive and deteriorating conditions. This research could inform the development of contextualised clinical guidelines and advocate for the training needs of wheelchair users from all populations and contexts.

## AUTHOR CONTRIBUTIONS

The authors of this research paper declare that the contributions to this work are as indicated below. Kimberly Charlton was responsible for Conceptualisation, Formal analysis, Methodology, and Writing—original draft, editing, and review. Carolyn Murray, Natasha Layton, and Stacie Attrill were responsible for Conceptualisation, Formal analysis, Methodology, Supervision, and Writing—review & editing. Emilee Ong, Lucy Farrar, and Trish Serocki were responsible for screening of studies and review and editing of writing. Each author has reviewed and approved the final version of the manuscript and agrees to be accountable for all aspects of the work.

## CONFLICT OF INTEREST STATEMENT

Dr Carolyn Murray is an Associate Editor for AOTJ and a co‐author of this article. C.M. was anonymized to the peer review process; management of the peer review process and decision‐making for this article was undertaken by the Editor‐in‐Chief.

## Supporting information


**Data S1.** Search Strategy.

**Data S2.** Study characteristics.

## Data Availability

Data sharing is not applicable to this article as no new data were created or analyzed in this study.
