# Supplementary material for: Manual wheelchair training approaches and intended training outcomes for adults who are new to wheelchair use: A scoping review
Source: Aust Occup Ther J. 2024 Oct 1;72(1):e12992. doi: 10.1111/1440-1630.12992 (PMC11649964; doi:10.1111/1440-1630.12992)
Supplement: Supplementary file 1 — Data S1. Search Strategy. [file AOT-72-0-s001.docx]

Supplementary material 1. Systematic Search Strategy

**EMBASE**

Date of Search: 28/07/2022

| **Search No.** | **Search Terms** | **Hits** |
| --- | --- | --- |
| 1 | wheelchair/ | 10607 |
| 2 | ("Wheel?chair*" or "chair?based" or "manual wheelchair").ti,ab,kf. | 12442 |
| 3 | education/ | 455767 |
| 4 | health education/ | 103750 |
| 5 | patient education/ | 120965 |
| 6 | practice guideline/ | 505819 |
| 7 | clinical trial protocol/ | 2006 |
| 8 | protocol compliance/ | 18546 |
| 9 | (educat* or train* or "health educat*" or instruction* or coach* or mentor*  or guideline* or "competency?based education" or "clinical guideline*"  or protocol* or "practice pathway").ti,ab,kf. | 2993861 |
| 10 | motor performance/ | 84820 |
| 11 | daily life activity/ | 104891 |
| 12 | (skill* or "skill?training" or "motor?skill*" or "wheelchair?skill* training"  or adl or "activit* of daily living" or "daily living activit*" or function*  or independen* or abilit*).ti,ab,kf. | 7697980 |
| 13 | 1 or 2 | 15318 |
| 14 | 3 or 4 or 5 or 6 or 7 or 8 or 9 | 3442123 |
| 15 | 10 or 11 or 12 | 7760061 |
| 16 | 13 and 14 and 15 | 1774 |

Search re-run : 1/9/2023

| **Search No.** | **Search Terms** | **Hits** |
| --- | --- | --- |
| 1 | wheelchair/ | 11563 |
| 2 | ("Wheel?chair*" or "chair?based" or "manual wheelchair").ti,ab,kf. | 13273 |
| 3 | education/ | 486484 |
| 4 | health education/ | 109330 |
| 5 | patient education/ | 125473 |
| 6 | practice guideline/ | 554724 |
| 7 | clinical trial protocol/ | 3111 |
| 8 | protocol compliance/ | 20869 |
| 9 | (educat* or train* or "health educat*" or instruction* or coach* or mentor* or guideline* or "competency?based education" or "clinical guideline*" or protocol* or "practice pathway").ti,ab,kf. | 3285719 |
| 10 | motor performance/ | 91394 |
| 11 | daily life activity/ | 114620 |
| 12 | (skill* or "skill?training" or "motor?skill*" or "wheelchair?skill* training" or adl or "activit* of daily living" or "daily living activit*" or function* or independen* or abilit*).ti,ab,kf. | 8278134 |
| 13 | 1 or 2 | 16555 |
| 14 | 3 or 4 or 5 or 6 or 7 or 8 or 9 | 3753075 |
| 15 | 10 or 11 or 12 | 8345191 |
| 16 | 13 and 14 and 15 | 1936 |
| 17 | limit 16 to yr="2022 - 2024" | 246 |

**COCHRANE LIBRARY**

Date of Search: 28/7/2022

| **Search No** | **Search Terms** | **Hits** |
| --- | --- | --- |
| #1 | MeSH descriptor: [Wheelchairs] explode all trees | 219 |
| #2 | ("Wheelchair*" or "chairbased" or "manual wheelchair"):ti,ab,kw | 879 |
| #3 | MeSH descriptor: [Education] explode all trees | 35711 |
| #4 | MeSH descriptor: [Patient Education as Topic] explode all trees | 9292 |
| #5 | MeSH descriptor: [Practice Guideline] explode all trees | 0 |
| #6 | MeSH descriptor: [Clinical Trial Protocol] explode all trees | 0 |
| #7 | MeSH descriptor: [Guideline Adherence] explode all trees | 1129 |
| #8 | MeSH descriptor: [Competency-Based Education] explode all trees | 89 |
| #9 | (educat* or train* or "health educat*" or instruction* or coach*  or mentor* or guideline* or "competency based education" or  "clinical guideline*" or protocol* or "practice pathway"):ti,ab,kw | 354933 |
| #10 | MeSH descriptor: [Motor Skills] explode all trees | 2067 |
| #11 | MeSH descriptor: [Activities of Daily Living] explode all trees | 10313 |
| #12 | (skill* or "skill training" or "motor skill*" or "wheelchair skill* training"  or adl or "activit* of daily living" or "daily living activit*" or function*  or independen* or abilit*):ti,ab,kw | 420580 |
| #13 | #1 OR #2 | 894 |
| #14 | #3 OR #4 OR #5 OR #6 OR #7 OR #8 OR #9 | 361709 |
| #15 | #10 OR #11 OR #12 | 424984 |
| #16 | #13 AND #14 AND #15 | 300 |

Search re-run: 1/9/2023

| **Search No.** | **Search Terms** | **Hits** |
| --- | --- | --- |
| #1 | MeSH descriptor: [Wheelchairs] explode all trees | 247 |
| #2 | ("Wheelchair" or "chairbased" or "manual wheelchair"):ti,ab,kw | 979 |
| #3 | MeSH descriptor: [Education] explode all trees | 43463 |
| #4 | MeSH descriptor: [Patient Education as Topic] explode all trees | 10091 |
| #5 | MeSH descriptor: [Practice Guideline] explode all trees | 1015 |
| #6 | MeSH descriptor: [Clinical Trial Protocol] explode all trees | 128 |
| #7 | MeSH descriptor: [Guideline Adherence] explode all trees | 1383 |
| #8 | MeSH descriptor: [Competency-Based Education] explode all trees | 99 |
| #9 | (educat* or train* or "health educat*" or instruction* or coach* or mentor* or guideline* or "competency based education" or "clinical guideline*" or protocol* or "practice pathway"):ti,ab,kw | 402139 |
| #10 | MeSH descriptor: [Motor Skills] explode all trees | 2276 |
| #11 | MeSH descriptor: [Activities of Daily Living] explode all trees | 13068 |
| #12 | (skill* or "skill training" or "motor skill*" or "wheelchair skill* training" or adl or "activit* of daily living" or "daily living activit*" or function* or independen* or abilit*):ti,ab,kw | 467111 |
| #13 | #1 OR #2 | 996 |
| #14 | #3 OR #4 OR #5 OR #6 OR #7 OR #8 OR #9 | 410328 |
| #15 | #10 OR #11 OR #12 | 472356 |
| #16 | #13 AND #14 AND #15 with Cochrane Library publication date Between Aug 2022 and Sep 2023 | 39 |

**EMCARE**

Date of Search: 28/07/2022

| **Search no.** | **Search Terms** | **Hits** |
| --- | --- | --- |
| 1 | wheelchair/ | 4412 |
| 2 | ("Wheelchair*" or "chair based" or "manual wheelchair").ti,ab,kf. | 5445 |
| 3 | education/ | 180926 |
| 4 | health education/ | 41204 |
| 5 | patient education/ | 42518 |
| 6 | practice guideline/ | 177958 |
| 7 | clinical trial protocol/ | 399 |
| 8 | protocol compliance/ | 6435 |
| 9 | (educat* or train* or "health educat*" or instruction* or coach*  or mentor* or guideline* or "competency?based education" s or "clinical guideline*" or protocol* or "practice pathway").ti,ab,kf. | 1027184 |
| 10 | motor performance/ | 27862 |
| 11 | daily life activity/ | 42850 |
| 12 | (skill* or "skill training" or "motor skill*" or  "wheelchair skill* training" or adl or "activit* of daily living"  or "daily living activit*" or function* or independen* or abilit*).ti,ab,kf. | 1482829 |
| 13 | 1 or 2 | 6313 |
| 14 | 3 or 4 or 5 or 6 or 7 or 8 or 9 | 1162126 |
| 15 | 10 or 11 or 12 | 1503873 |
| 16 | 13 and 14 and 15 | 884 |
|  | Search re-run: 1/9/2023 (From 2022- current)   \| **Search no.** \| **Search Terms** \| **Hits** \| \| --- \| --- \| --- \| \|  \|  \|  \| \| 1 \| wheelchair/ \| 4121 \| \| 2 \| ("Wheelchair*" or "chair based" or "manual wheelchair").ti,ab,kf. \| 5859 \| \| 3 \| education/ \| 178161 \| \| 4 \| health education/ \| 34653 \| \| 5 \| patient education/ \| 35895 \| \| 6 \| practice guideline/ \| 138674 \| \| 7 \| clinical trial protocol/ \| 280 \| \| 8 \| protocol compliance/ \| 3537 \| \| 9 \| (educat* or train* or "health educat*" or instruction* or coach* or mentor* or guideline* or "competency?based education" or "clinical guideline*" or protocol* or "practice pathway").ti,ab,kf. \| 1142778 \| \| 10 \| motor performance/ \| 22821 \| \| 11 \| daily life activity/ \| 34551 \| \| 12 \| (skill* or "skill training" or "motor skill*" or "wheelchair skill* training" or adl or "activit* of daily living" or "daily living activit*" or function* or independen* or abilit*).ti,ab,kf. \| 1633873 \| \| 13 \| 1 or 2 \| 6590 \| \| 14 \| 3 or 4 or 5 or 6 or 7 or 8 or 9 \| 1246246 \| \| 15 \| 10 or 11 or 12 \| 1650063 \| \| 16 \| 13 and 14 and 15 \| 930 \| \| 17 \| limit 16 to yr="2022 -Current" \| 104 \| \| 18 \| 16 and 17 \| 104 \| |  |

**MEDLINE**

Date of Search: 28/07/2022

| **Search No.** | **Search Terms** | **Hits** |
| --- | --- | --- |
| 1 | Wheelchairs/ | 5259 |
| 2 | ("Wheel?chair*" or "chair?based" or "manual wheelchair").ti,ab,kf. | 8294 |
| 3 | Education/ | 21503 |
| 4 | Health Education/ | 63063 |
| 5 | Patient Education as Topic/ | 88108 |
| 6 | Guideline/ | 16527 |
| 7 | Practice Guideline/ | 29969 |
| 8 | Clinical Trial Protocol/ | 9310 |
| 9 | Guideline Adherence/ | 34807 |
| 10 | Competency-Based Education/ | 4471 |
| 11 | (educat* or train* or "health educat*" or instruction* or coach* or mentor* or guideline*  or "competency?based education" or "clinical guideline*" or protocol* or  "practice pathway").ti,ab,kf. | 2190417 |
| 12 | Motor Skills/ | 26100 |
| 13 | Activities of Daily Living/ | 70992 |
| 14 | (skill* or "skill?training" or "motor?skill*" or "wheelchair?skill* training" or adl  or "activit* of daily living" or "daily living activit*" or function* or independen*  or abilit*).ti,ab,kf. | 6191550 |
| 15 | 1 or 2 | 9449 |
| 16 | 3 or 4 or 5 or 6 or 7 or 8 or 9 or 10 or 11 | 2304326 |
| 17 | 12 or 13 or 14 | 6226803 |
| 18 | 15 and 16 and 17 | 1062 |

Search re-run: 1/9/2023

| **Search No.** | **Search Terms** | **Hits** |
| --- | --- | --- |
|  |  |  |
| 1 | Wheelchairs/ | 5417 |
| 2 | ("Wheel?chair*" or "chair?based" or "manual wheelchair").ti,ab,kf. | 8774 |
| 3 | Education/ | 21528 |
| 4 | Health Education/ | 63747 |
| **5** | Patient Education as Topic/ | 88317 |
| 6 | Guideline/ | 16582 |
| 7 | Practice Guideline/ | 30617 |
| 8 | Clinical Trial Protocol/ | 11260 |
| 9 | Guideline Adherence/ | 35093 |
| 10 | Competency-Based Education/ | 4619 |
| **11** | (educat* or train* or "health educat*" or instruction* or coach* or mentor* or guideline* or "competency?based education" or "clinical guideline*" or protocol* or "practice pathway").ti,ab,kf. | 2386941 |
| 12 | Motor Skills/ | 26465 |
| 13 | Activities of Daily Living/ | 73189 |
| 14 | (skill* or "skill?training" or "motor?skill*" or "wheelchair?skill* training" or adl or "activit* of daily living" or "daily living activit*" or function* or independen* or abilit*).ti,ab,kf. | 6601858 |
| 15 | 1 or 2 | 9930 |
| 16 | 3 or 4 or 5 or 6 or 7 or 8 or 9 or 10 or 11 | 2501285 |
| 17 | 12 or 13 or 14 | 6637225 |
| 18 | 15 and 16 and 17 | 1150 |
| 19 | limit 18 to yr="2022 -Current" | 159 |

**PUB MED**

Date of Search: 27/07/2022

| **Search no.** | **Search Terms** | **Hits** |
| --- | --- | --- |
| 15 | (((wheelchairs[MeSH Terms]) OR (wheelchair[Title/Abstract]  OR chair based[Title/Abstract] OR "manual wheelchair"[Title/Abstract]))  AND (((((("educat*"[Title/Abstract] OR "train*"[Title/Abstract] OR  "health educat*"[Title/Abstract] OR "instruction*"[Title/Abstract] OR  "coach*"[Title/Abstract] OR "mentor*"[Title/Abstract] OR "guideline*"[Title/Abstract] OR "competency-based education"[Title/Abstract] OR "clinical guideline*"[Title/Abstract] OR "protocol*"[Title/Abstract] OR "practice pathway"[Title/Abstract])) OR (competency based education[MeSH Terms])) OR (guideline adherence[MeSH Terms])) OR (health education[MeSH Terms])) OR (education[MeSH Terms]))) AND ((("skill*"[Title/Abstract] OR "skill training"[Title/Abstract] OR "motor skill*"[Title/Abstract] OR "adl"[Title/Abstract] OR "daily living activities"[Title/Abstract] OR "function*"[Title/Abstract] OR "independen*"[Title/Abstract] OR "abilit*"[Title/Abstract] OR "wheelchair skills training"OR "activities of daily living"[Title/Abstract]) OR (motor skills[MeSH Terms])) OR (activities of daily living[MeSH Terms])) | 1,075 |
| 14 | (("skill*"[Title/Abstract] OR "skill training"[Title/Abstract] OR  "motor skill*"[Title/Abstract] OR "adl"[Title/Abstract] OR "daily living activities"[Title/Abstract] OR "function*"[Title/Abstract] OR "independen*"[Title/Abstract] OR "abilit*"[Title/Abstract] OR "wheelchair skills training"OR "activities of daily living"[Title/Abstract]) OR (motor skills[MeSH Terms])) OR (activities of daily living[MeSH Terms]) | 6,240,479 |
| 13 | ((((("educat*"[Title/Abstract] OR "train*"[Title/Abstract] OR  "health educat*"[Title/Abstract] OR "instruction*"[Title/Abstract] OR "coach*"[Title/Abstract] OR "mentor*"[Title/Abstract] OR "guideline*"[Title/Abstract] OR "competency-based education"[Title/Abstract] OR "clinical guideline*"[Title/Abstract] OR "protocol*"[Title/Abstract] OR "practice pathway"[Title/Abstract])) OR (competency based education[MeSH Terms])) OR (guideline adherence[MeSH Terms])) OR (health education[MeSH Terms])) OR (education[MeSH Terms]) | 2,691,603 |
| 12 | (wheelchairs[MeSH Terms]) OR (wheelchair[Title/Abstract]  OR chair based[Title/Abstract] OR "manual wheelchair"[Title/Abstract]) | 9,095 |
| 11 | activities of daily living[MeSH Terms] | 116,003 |
| 10 | motor skills[MeSH Terms] | 26,076 |
| 9 | "skill*"[Title/Abstract] OR "skill training"[Title/Abstract] OR "motor skill*" [Title/Abstract] OR "adl"[Title/Abstract] OR "daily living activities"[Title/Abstract] OR "function*"[Title/Abstract] OR "independen*"[Title/Abstract] OR "abilit*"[Title/Abstract] OR "wheelchair skills training"OR "activities of daily living"[Title/Abstract] | 6,173,355 |
| 8 | education[MeSH Terms] | 923,896 |
| 7 | health education[MeSH Terms] | 258,977 |
| 6 | guideline adherence[MeSH Terms] | 34,786 |
| 5 | competency based education[MeSH Terms] | 4,464 |
| 4 | "educat*"[Title/Abstract] OR "train*"[Title/Abstract] OR "health educat* "[Title/Abstract] OR "instruction*"[Title/Abstract] OR "coach*"[Title/Abstract] OR "mentor*"[Title/Abstract] OR "guideline*"[Title/Abstract] OR "competency-based education"[Title/Abstract] OR "clinical guideline*"[Title/Abstract] OR "protocol*"[Title/Abstract] OR "practice pathway"[Title/Abstract] | 2,180,464 |
| 3 | wheelchair[Title/Abstract] OR chair based[Title/Abstract] OR  "manual wheelchair"[Title/Abstract] | 7,685 |
| 2 | wheelchairs[MeSH Terms] | 5,235 |
| 1 | "motor skills"[mh] OR "activities of daily living"[mh] OR "skill* "[Title/Abstract] OR "skill training"[Title/Abstract] OR "motor skill*"[Title/Abstract] OR "adl"[Title/Abstract] OR "daily living activities"[Title/Abstract] OR "function*"[Title/Abstract] OR "independen*"[Title/Abstract] OR "abilit*"[Title/Abstract] OR "wheelchair skills training"[Title/Abstract] OR "activities of daily living"[Title/Abstract] AND "education"[mh] OR "health education"[mh] OR "guideline adherence"[mh] OR "competency-based education"[mh] OR educat*[Title/Abstract] OR train*[Title/Abstract] OR "health educat*"[Title/Abstract] OR instruction*[Title/Abstract] OR coach*[Title/Abstract] OR mentor*[Title/Abstract] OR guideline*[Title/Abstract] OR "competency based education"[Title/Abstract] OR "clinical guideline*"[Title/Abstract] OR protocol*[Title/Abstract] OR "practice pathway"[Title/Abstract] AND "wheelchairs" [mh] OR Wheelchair*[Title/Abstract] OR chair based[Title/Abstract] OR manual wheelchair*[Title/Abstract] - Saved search | 8,568 |

Search re-run: 1/9/2023 (Date range (1/8/2022- 01/09/2023)

| **Search no.** | **Search Terms** | **Hits** |
| --- | --- | --- |
| 1 | Search: "motor skills"[mh] OR "activities of daily living"[mh] OR "skill* "[Title/Abstract] OR "skill training"[Title/Abstract] OR "motor skill*"[Title/Abstract] OR "adl"[Title/Abstract] OR "daily living activities"[Title/Abstract] OR "function*"[Title/Abstract] OR "independen*"[Title/Abstract] OR "abilit*"[Title/Abstract] OR "wheelchair skills training"[Title/Abstract] OR "activities of daily living"[Title/Abstract] AND "education"[mh] OR "health education"[mh] OR "guideline adherence"[mh] OR "competency-based education"[mh] OR educat*[Title/Abstract] OR train*[Title/Abstract] OR "health educat*"[Title/Abstract] OR instruction*[Title/Abstract] OR coach*[Title/Abstract] OR mentor*[Title/Abstract] OR guideline*[Title/Abstract] OR "competency based education"[Title/Abstract] OR "clinical guideline*"[Title/Abstract] OR protocol*[Title/Abstract] OR "practice pathway"[Title/Abstract] AND "wheelchairs" [mh] OR Wheelchair*[Title/Abstract] OR chair based[Title/Abstract] OR manual wheelchair*[Title/Abstract] Filters: from 2022/8/1 - 2023/9/1 | 5232 |

**CINAHL**

Date of Search: 28/07/2022

| **Search no.** | **Search Terms** | **Hits** |  |
| --- | --- | --- | --- |
| S15 | S12 AND S13 AND S14 | 742 |  |
|  |  |  |  |
| S14 | S9 OR S10 OR S11 | 1,000,465 |  |
|  |  |  |  |
| S13 | S3 OR S4 OR S5 OR S6 OR S7 OR S8 | 1,153,948 |  |
|  |  |  |  |
| S12 | S1 OR S2 | 8,491 |  |
|  |  |  |  |
| S11 | ( TI (skill* OR "skill training" OR "motor skill*" OR "wheelchair skill* training" OR adl OR "activit* of daily living" OR "daily living activit*" OR function* OR independen* OR abilit*) ) OR ( AB (skill* OR "skill training" OR "motor skill*" OR "wheelchair skill* training" OR adl OR "activit* of daily living" OR "daily living activit*" OR function* OR independen* OR abilit*) ) | 981,758 |  |
| S10 | (MH "Activities of Daily Living") OR (MH "Instrumental Activities of Daily Living Alteration (Saba CCC)") OR (MH "Instrumental Activities of Daily Living (Saba CCC)") OR (MH "Activities of Daily Living Alteration (Saba CCC)") OR (MH "Activities of Daily Living (Saba CCC)") OR (MH "Self Care: Activities of Daily Living (Iowa NOC)") OR (MH "Altered Activities of Daily Living (NANDA)") | 36,519 |  |
| S9 | (MH "Motor Skills") | 12,139 |  |
|  |  |  |  |
| S8 | ( TI (educat* OR train* OR "health educat*" OR instruction* OR coach* OR mentor* OR guideline* OR "competency based education" OR "clinical guideline*" OR protocol* OR practice pathway) ) OR ( AB (educat* OR train* OR "health educat*" OR instruction* OR coach* OR mentor* OR guideline* OR "competency based education" OR "clinical guideline*" OR protocol* OR practice pathway) ) | 915,479 |  |
| S7 | (MH "Education, Competency-Based") | 3,282 |  |
|  |  |  |  |
| S6 | (MH "Clinical Trials") | 183,855 |  |
|  |  |  |  |
| S5 | (MH "Practice Guidelines") | 84,857 |  |
|  |  |  |  |
| S4 | (MH "Education, Allied Health") | 1,613 |  |
|  |  |  |  |
| S3 | (MH "Patient Education") | 69,559 |  |
|  |  |  |  |
| S2 | TI ("Wheelchair*" OR "chair?based" OR "manual wheelchair") OR AB ("Wheelchair*" OR "chair?based" OR "manual wheelchair") | 6,395 |  |
| S1 | MH "wheelchairs" | 5,377 |  |
|  |  |  |  |

Search re-run: 1/9/2023 (August 2023- September 2024)

**PROQUEST**

Date of Search- 28/07/2022

| **Search No.** | **Search** | **Hits** |
| --- | --- | --- |
|  | [abstract("Wheelchair*" OR "chair?based" OR "manual wheelchair") AND abstract(educat* OR train* OR "health educat*" OR instruction* OR coach* OR mentor* OR guideline* OR "competency based education" OR "clinical guideline*" OR protocol* OR practice pathway) AND abstract(skill* OR "skill training" OR "motor skill*" OR "wheelchair skill* training" OR adl OR "activit* of daily living" OR "daily living activit*" OR function* OR independen* OR abilit*) AND pd(>20001231)](https://www.proquest.com/myresearch/savedsearches.checkdbssearchlink:rerunsearch/2251733/SavedSearches?t:ac=SavedSearches) | 732 |

Search re-run: 1/9/23 (From 1/8/22- current)

| **Search No.** | **Search** | **Hits** |  |  |  |  |  |
| --- | --- | --- | --- | --- | --- | --- | --- |
|  | [abstract("Wheelchair*" OR "chair?based" OR "manual wheelchair") AND abstract(educat* OR train* OR "health educat*" OR instruction* OR coach* OR mentor* OR guideline* OR "competency based education" OR "clinical guideline*" OR protocol* OR practice pathway) AND abstract(skill* OR "skill training" OR "motor skill*" OR "wheelchair skill* training" OR adl OR "activit* of daily living" OR "daily living activit*" OR function* OR independen* OR abilit*)Limits applied](https://www.proquest.com/recentsearches.recentsearchtabview.recentsearchesgridview.scrolledrecentsearchlist.checkdbssearchlink:rerunsearch/3E40D39057154982PQ/None?t:ac=RecentSearches) | 37 |  |  |  |  |  |

**SCOPUS**

Date of Search – 2/07/2022

| **Search No.** | **Search Terms** | **hits** |
| --- | --- | --- |
| 1 | ( TITLE-ABS-KEY ( wheelchair*  OR  chair  AND based  OR  manual  AND wheelchair ) )  AND  ( TITLE-ABS-KEY ( educat*  OR  train*  OR  "health educat*"  OR  instruction*  OR  coach*  OR  mentor*  OR  guideline*  OR  "competency based education"  OR  "clinical guideline*"  OR  protocol*  OR  "practice pathway" ) )  AND  ( TITLE-ABS-KEY ( skill*  OR  "skill training"  OR  "motor skill*"  OR  "wheelchair skill* training"  OR  adl  OR  "activit* of daily living"  OR  "daily living activit*"  OR  function*  OR  independen*  OR  abilit* ) ) | 782 |

Search re-run: 1/9/2023 (2022-2023)

| **Search No.** | **Search Terms** | **hits** |
| --- | --- | --- |
| 1 | (TITLE-ABS-KEY(wheelchair* OR chair based OR manual wheelchair)) AND (TITLE-ABS-KEY(educat* OR train* OR "health educat*" OR instruction* OR coach* OR mentor* OR guideline* OR "competency based education" OR "clinical guideline*" OR protocol* OR "practice pathway")) AND (TITLE-ABS-KEY(skill* OR "skill training" OR "motor skill*" OR "wheelchair skill* training" OR adl OR "activit* of daily living" OR "daily living activit*" OR function* OR independen* OR abilit*)) AND PUBYEAR > 2021 AND PUBYEAR < 2024 | 124 |
