# Supplementary material for: Manual wheelchair training approaches and intended training outcomes for adults who are new to wheelchair use: A scoping review
Source: Aust Occup Ther J. 2024 Oct 1;72(1):e12992. doi: 10.1111/1440-1630.12992 (PMC11649964; doi:10.1111/1440-1630.12992)
Supplement: Supplementary file 2 — Data S2. Study characteristics. [file AOT-72-0-s002.docx]

Supplementary file 2. Study Characteristics table

| Author/Date | Type of Paper | Main aim of Paper |
| --- | --- | --- |
| Arlati et al., 2018 | Participatory action design | To design a framework aimed at supporting novice wheelchair users return to work and activities of daily living. |
| Beaudoin et al., 2021 | Mixed Methods | To measure the influence of Roulez Avec Confidence MWC training program on satisfaction with participation, MWC use self-efficacy, MWC skills capacity, performance and quality of life. |
| Best et al., 2005 | RCT | To determine if wheelchair skills training of community-based wheelchair users is efficacious, safe, and practical. |
| Best et al., 2015 | Survey | To describe current practices for MWC skills training in Canadian rehabilitation centres. |
| Best et al., 2016 | RCT | To evaluate the effect of a peer-led wheelchair training program on self-efficacy of MWC use, MWC skills, life-space mobility, and satisfaction with participation. |
| Blouin et al., 2015 | Cohort Study | To investigate whether haptic biofeedback can lead MWC users to modify their propulsive force and propulsive patterns. |
| Bonaparte et al., 2004 | RCT | To determine whether adding the proactive balance strategy (PBS) to the conventional reactive balance strategy (RBS) increases the success rate, decreases training time, and lessens postural sway during the learning of the wheelie skill. |
| Budai & Murdoch 2019 | Summary | To provide a summary of wheelchair training considerations. |
| Caro & Cruz 2020 | Literature Review | To map the interventions for manual wheelchairs skills training available in the literature of the last 10 years. |
| Chaar & Archambault, 2022 | Qualitative Research | To assess the usability and fidelity of the virtual reality simulator, by clinicians and expert MWC users and if haptic feedback influences users’ experiences |
| Charbonneau et al., 2013 | Cross Sectional Study | Explore the merits of forward versus backward wheelchair propulsion in WC users with hemiplegia. |
| Charlton et al., 2021 | Mixed Methods | To understand the outcomes for people with lower limb amputation participating in group WC training and the perspectives of participants and facilitators about wheelchair training within an inpatient rehabilitation setting. |
| Chen 2019 | RCT | Examine the efficacy of a repetition-based wheelchair propulsion training program for teaching clinical practice guidelines. |
| Choi et al., 2020 | Cross Sectional Study | To develop a wheelchair training structure as an adjustable type and verify its usefulness. |
| Cooper et al., 2005 | Summary Paper | To provide a summary of research regarding the use of Virtual reality for mobility skill assessment and training. |
| Ctri 2023 | Protocol | To compare the effectiveness of 3 types of wheelchair skills training. |
| DeGroot et al., 2009 | Cohort Study | To determine if verbal training with visual feedback improves MWC propulsion. |
| Denison, 2013 | Training Program | To provide an instructional manual for teaching people to use their wheelchair |
| Desai et al., 2013 | Pre and Post design | To explore the immediate and short-term effect of MWC skills training and its effect on participation in persons with Spinal Cord Injury. |
| Furmaniuk et al., 2010 | Pre and Post design | To assess the influence of long-term wheelchair rugby training on the functional abilities of persons with tetraplegia over a 2-year period post-spinal cord injury |
| Garrett et al., 2011 | Case Study | Outline the the Wheelchair Skills Program testing and training methods as well as wheelchair maintenance. |
| Genova et al., 2022 | Summary Paper | To describe simulation models for both manual and powered wheelchair based on immersive virtual reality (CAVE). |
| Giesbrecht & Miller 2019 | RCT | To evaluate the impact of an mHealth wheelchair skills training program (EPIC Wheels) on clinical outcomes among older adult MWC users. |
| Giesbrecht et al., 2014 | Qualitative Research | To develop a prototype Wheelchair Skills Training Program (EPIC wheels) that could be delivered as a home program using a computer tablet |
| Giesbrecht et al., 2015 | Summary Paper | To provide a summary of the MWC training programs: EPIC Wheels (Enhancing Participation In the Community by improving Wheelchair Skills) and WheelSeeU (Wheelchair training Self-efficacy enhanced for Use). |
| Giesbrecht et al., 2015b | Pre and Post design | To determine the acceptability and feasibility of administering an mHealth wheelchair skills training program (EPIC skills) safely and effectively. |
| Giesbrecht et al., 2015c | Qualitative Research | To develop an understanding of the experience transitioning to wheelchair use for older adult users and their care providers. |
| Giesbrecht et al., 2017 | RCT | To evaluate the feasibility of implementing an RCT with an mHealth MWC skills training program among middle-aged and older adults. |
| Giesbrecht et al., 2021 | Protocol | To outline implementation and evaluation of the TEAM Wheels training program in a randomized control trial. |
| Keeler et al., 2019 | Systematic Review | To conduct a systematic review synthesizing the evidence for the effectiveness of the Wheelchair Skills Training Program. |
| Kehrer et al., 2021 | Systematic Review | To examine the effectiveness of rehabilitation interventions for middle-aged People ageing with long term physical disabilities to participate independently in the home and community. |
| Kirby et al., 2001 | RCT | To evaluate the safety and efficacy of a wheelie aid. |
| Kirby et al., 2004 | Pre and Post design | To test the hypothesis that the Wheelchair skills training program is effective in improving the wheel-chair-handling skills of untrained caregivers. |
| Kirby et al., 2006 | Literature Review | To review the different rear wheelie training methods performed by WC users. |
| Kirby et al., 2008 | RCT | To explore the efficacy and safety of conventional rear anti-tip devices (CRADs) compared to those using a new RAD design that deploy through an arc (ArcRAD) |
| Kirby et al., 2015 | Survey | To determine the proportion of wheelchair users who receive MWC skills training during an inpatient stay at a Canadian rehabilitation centre. |
| Kirby et al., 2016 | RCT | To evaluate whether community-dwelling veterans with spinal cord injury who receive the Wheelchair Skills Training Program (WSTP) improve their manual wheelchair-skills capacity and participation. |
| Kirby et al., 2020 | Survey | To determine the extent, nature and perceptions of training wheelchair skills training of Occupational Therapists in Nova Scotia. t |
| Kirby et al., 2021 | Survey | To determine the extent to which wheelchair service providers conduct wheelchair-skills training, the nature and the perceptions of training. |
| Kotajarvi et al., 2006 | Cohort Study | To determine the effect of visual feedback on the propulsion effectiveness of experienced manual wheelchair users |
| Lam et al., 2018 | Systematic Review | To provide a description of the current knowledge regarding the use of virtual technology for wheelchair skills training. |
| Limroongreungrat et al., 2009 | RCT | To examine effects of real-time video feedback on level wheelchair propulsion training for people with SCI |
| Liu et al., 2019 | Qualitative Research | To provide perspectives on the useability of My Wheelchair Guide app. |
| MacPhee, 2004 | RCT | To develop and evaluate a wheelchair skills training protocol incorporating motor-learning principles into a rehabilitation setting. |
| Mathias et al., 2021 | Survey | To gain understanding how Irish health professionals offer MWC training. |
| McClure et al., 2010 | RCT | To describe the development of a strict education protocol to implement the clinical practice guideline "Preservation of Upper Limb Function Following Spinal Cord Injury" and evaluate manual wheelchair propulsion of individuals with new spinal cord injuries who have been strictly educated on the clinical practice guideline. |
| Michael, 2013 | Text/opinion paper | To provide an overview of wheelchair training skills that can support client independence. |
| Miller et al., 2019 | RCT | To explore the efficacy of a peer-led Wheelchair Self-Efficacy Enhanced for Use (WheelSeeU) program on wheelchair skills, wheelchair use self-efficacy, satisfaction with participation, life-space mobility, and participation frequency. |
| Morgan et al., 2017 | Qualitative Research | To identify wheelchair skills currently being taught to new manual wheelchair users and identify similarities and differences between the perspectives of health care professionals and manual wheelchair users. |
| Morgan et al., 2017 | Pre and Post design | To pilot manual wheelchair training based on motor learning and repetition-based approaches for new manual wheelchair users with SCI. |
| North Western Regional Spinal Cord Injury System, 2021 | Training Program | A guide learning the proper techniques for performing manual wheelchair skills, managing different kinds of terrain, and transferring to and from a wheelchair. |
| Ozturk et al., 2011 | RCT | To determine if community-dwelling wheelchair users in Turkey who have completed the Wheelchair Skills Training Program have better total percentage performance and safety scores on the Wheelchair Skills Test. |
| Park & Jung 2022 | RCT | To evaluate the modified WSP for improving wheelchair skills capacity, perceived satisfaction and performance in daily activities for hemiplegia patients in Korea. |
| Pellichero 2020 | Qualitative Research | To explore older adults' perceptions and experiences about their participation in the WheelSeeU program. |
| Physiopedia | Training Program | To provide comprehensive knowledge of the importance of adequate user fitting and training including both specific wheelchair and maintenance skills |
| Pouvrasseau et al., 2017 | Qualitative Research | To discuss the Virtual Fauteuil platform, which offers simulation of travel with a wheelchair in a virtual environment. |
| Push Mobility, 2023 | Training Program | To provide knowledge of skills for independent and safe wheelchair use. |
| Quinones-Uriostegui et al., 2017 | Pre and Post design | To assess the WHO 8 step guidelines on the provision of manual wheelchairs in less resourced settings on user satisfaction, wheelchair skills and quality of life of Mexican MWC users. |
| Rahim et al., 2021 | Scoping Review | To identify the types of available evidence on wheelchair skills training among People with Disability. |
| Requejo et al., 2008 | Summary Paper | To present current available evidence and recommendations for preserving function and mobility for elderly and aging individuals using an MWC. |
| Rice & Rice 2017 | Literature Review | To examine education interventions to preserve upper limb function and prevent the development of pain and injury among full time MWC users. |
| Rice et al., 2010 | Case Study | The objective of this study was to describe the development of a manual wheelchair propulsion training program aimed to promote the development of an efficient propulsion technique among long-term manual wheelchair users. |
| Rice et al., 2013 | RCT | To compare the effects of 2 manual wheelchair propulsion training programs on hand rim kinetics, contact angle, and stroke frequency. |
| Rice et al., 2014 | RCT | To determine if strict use of the Paralysed Veterans of America's Clinical Practice Guidelines for Preservation of Upper Limb Function affects wheelchair setup, selection, propulsion biomechanics, pain, satisfaction with life, and participation of individuals with new spinal cord injuries. |
| Rice et al., 2015 | RCT | To examine the efficacy and feasibility of a multifactorial intervention including propulsive skill and technique training based on the social cognitive theory on lifestyle physical activity in non-ambulatory persons with multiple sclerosis. |
| Richter et al., 2011 | Pre and Post design | To determine the ability of wheelchair users to make both large and small, targeted changes to select propulsion variables. |
| Robinson et al., 2022 | Training Program | To guide clinicians in a graded approach to wheelchair skills acquisition. |
| Rodgers et al., 2001 | Pre and Post design | To determine the effect of a specific training program in manual wheelchair users. |
| Routhier et al., 2012 | RCT | To determine the efficacy of the French-Canadian version of the Wheelchair Skills Training Program on wheelchair skills capacity. |
| Rusek et al., 2021 | Qualitative Research | To identify clinician knowledge regarding manual wheelchair training in an inpatient rehabilitation setting, identify current MWC education provided to new manual wheelchair users and determine how MWC training resources can be developed or modified to promote use among clinicians. |
| Sawatzky et al., 2015 | Position Paper | To examine the existing 2005 Clinical Practice Guidelines for preservation of the upper extremities in spinal cord injury and explore the literature to determine what might need to be included in an update. |
| Smith et al., 2009 | RCT | To evaluate in a Veterans long-term care setting, the efficacy of the Wheelchair Skills Training Program with manual wheelchair users. |
| Standal et al., 2008 | Qualitative Research | To investigate the learning that takes place when people with disabilities interact in a rehabilitation context. |
| Symonds et al., 2018 | Systematic Review | To examine whether real-time feedback can be used to influence manual wheelchair propulsion biomechanics |
| Tasiemski, et al., 2021 | Pre and Post design | To assess wheelchair skills of persons with physical disabilities attending the first Wheelchair Skills and Empowerment Camp in Morocco. |
| Taylor et al., 2015 | Cohort Study | To describe the type and quantity of MWC skills training provided by OTs and PTs during inpatient SCI rehabilitation. |
| The Back up Trust, 2022 | Training Program | To describe training for MWC skills in a hospital, training in real world settings, training at a designated residential training facility and training program via a wheelchair skills app that enables MWC users to practice MWC skills over a sort series of videos if not able to complete face to- face training. |
| Tu et al., 2017 | Systematic Review | To assess the effectiveness and safety of wheelchair skills training program in improving wheelchair skills capacity. |
| Van Der Scheer et al., 2015 | RCT | To investigate the effects of a low-intensity wheelchair training on propulsion technique in inactive people with long-term spinal cord injury. |
| Van Der Scheer et al., 2016 | RCT | To investigate the effects of low-intensity wheelchair training on wheelchair-specific fitness, wheelchair skill performance and physical activity levels in inactive people with long-term spinal cord injury. |
| WHO, 2012 | Training Program | To support the training of personnel fulfilling clinical and technical roles in a wheelchair service. The training package includes; how to train users and caregivers how to use and maintain the wheelchair. |
| WHO, 2013 | Training Program | To develop the skills and knowledge of personnel involved in wheelchair service delivery. Included in this training package is user training in Wheelchair Use. |
| WHO, 2017 | Training Program | To develop trainers to deliver training sessions, adult learning principles, presenting and facilitating, communication skills, managing group dynamics, audio visual tools. |
| Worobey et al., 2016 | RCT | To assess the effectiveness of group wheelchair skills training to elicit improvements in wheelchair skills. |
| Yeo & Kwon 2018 | RCT | To examine the effectiveness of wheelchair skills training in improving both wheelchair skills and upper arm skilled performance in adults with Cervical-SCI. |
| Yong Tai et al., 2015 | RCT | To examine the effectiveness of using immediate video feedback in a rehabilitation setting to train manual wheelchair users with SCI in learning three wheelchair skills. |
| Zwinkels et al., 2014 | Systematic Review | To review literature on the effectiveness of training programs in improving hand rim wheelchair propulsion capacity. |

Abbreviations: MWC = manual wheelchair; RCT = randomised control trial; SCI= spinal cord injury
